# Effects of COVID-19 lockdown on physical, mental and emotional parameters among sportspersons

**DOI:** 10.4102/hsag.v28i0.2119

**Published:** 2023-03-20

**Authors:** Amaarah Khan, Ammaarah Patel, Habib Noorbhai

**Affiliations:** 1 Department of Sport and Movement Studies, Faculty of Health Sciences, University of Johannesburg, Johannesburg, South Africa

**Keywords:** physical activity restriction, fitness, stress, well-being, athletes, COVID-19

## Abstract

**Background:**

The coronavirus disease 2019 (COVID-19) lockdown was a strange and new occurrence, which left many individuals ill-equipped to cope with the new way of living. Sportspersons had to adapt to a new training style within a new environment, both physically and mentally.

**Aim:**

The purpose of this study was to understand the physical, mental and emotional parameters among sportspersons during the COVID-19 lockdown regulations.

**Setting:**

The study consisted of 105 regular sportspersons (from South Africa).

**Methods:**

This was a quantitative research study design using an online questionnaire. An online questionnaire was adapted and distributed via online social platforms (WhatsApp, Twitter and Instagram) to collect data in which sportspersons (*n* = 105) answered questions about the effects that they experienced during lockdown on their physical, mental and emotional well-being.

**Results:**

Sportspersons participated in cardiovascular training, flexibility training, strength training and bodybuilding exercises during pre-lockdown. During lockdown, more than 74% of sportspersons had adequate training space, equipment and the time to perform physical activity. However, more than 43% of these sportspersons experienced a decrease in flexibility, muscle mass and muscle strength. Exercise was used as a form of stress relief by 77.1% of sportspersons throughout lockdown. In addition, sportspersons who used exercise as a form of stress relief continued to experience an increase in stress throughout lockdown.

**Conclusion:**

The outcomes from this study demonstrated how the COVID-19 lockdown had adverse effects on the overall health and well-being of most sportspersons. Other outcomes included the effects that physical inactivity had among sportspersons, including changes in diet and sleep.

**Contribution:**

This study highlights the urgency for the sports fraternity to adopt measures to provide various methods of stress relief (as well as opportunities for physical activity) during similar periods of lockdown (or exercise restrictions) for those who rely on exercise as their daily physical, mental and emotional outlet.

## Introduction

Earlier in March 2020, the World Health Organization (WHO) declared the coronavirus disease 2019 (COVID-19) outbreak as a global pandemic (Cucinotta & Vanelli 2020). Coronavirus disease 2019 was identified as the cause of an outbreak of respiratory illness first identified in Wuhan, China. This disease spread to South Africa where it was then announced as a national state of disaster (Parker et al. 2020). This resulted in the introduction of a national lockdown. These new regulations resulted in negative implications for many people, including the sporting fraternity, who regularly make use of public training facilities.

As a result, they were confined to their homes where they may have not been able to train as they ordinarily would. The closure of public facilities and safe training spaces resulted in these sportspersons altering their training methods to suit their current living arrangements. The COVID-19 lockdown was a strange and new occurrence, which left many individuals ill-equipped to cope with the new way of living. Sportspersons had to adapt to a new training style within a new environment, both physically and mentally. Regular sportspersons who worked out with partners, trainers or in training groups had to find ways to discipline themselves to continue training at home or risk losing muscle mass and muscle strength. The COVID-19 lockdown was a strange and new occurrence that left many individuals ill-equipped to cope with the new way of living. This study aimed to assess and create awareness about the extent of the physical and mental parameters on the average sportsperson. This study also addresses the effects of inactivity on the body.

## Methodology

### Study aim

The aim of this study was to determine the effects of the COVID-19 lockdown on the physical, mental and emotional parameters of regular sportspersons.

### Study design

This was a quantitative research study design using an online questionnaire.

### Study sample

The sample of the study was 105 regular sportspersons (from South Africa), which fall within the inclusion criteria of the study. This was calculated by using a 95% confidence level, a 9.8% margin of error, with a population proportion of 50%, and an unlimited population size. Physically active individuals who met the requirements of the study were invited to participate on their own free will, through an online questionnaire.

### Inclusion and exclusion criteria

- Regular sportspersons who exercised for more than 150 min per week consistently for over 3 months and who were within the range of 18–55 years of age were included in the study.
- All genders were included in this study.
- Sportspersons who suffered with a diagnosed mental disorder had not been included in the study.
- Sportspersons who had contracted the virus during the time frame but had recovered and returned to training were included in the study.

### Research instruments

A survey adapted from a Fitness and Wellness Questionnaire from Anand Medical Spa composed on Google Forms was used for this study. This questionnaire included questions about sportspersons’ physical and mental well-being throughout the COVID-19 lockdown period. These questions included a nominal scale as well as open-ended questions.

### Data collection

Questionnaires were distributed via an online link over social platforms (WhatsApp, Instagram, Twitter and via email), which directed sportspersons to the Google Forms questionnaire. These answers were exported through an automatically generated Microsoft Excel spreadsheet, which was then exported for analysis. The online questionnaire closed as soon as the target sample size was reached.

### Data analysis

Data analysis was conducted through a thematic analysis of open-ended questions using Microsoft Excel and was used to identify common themes that emerged from the data. The normality tests used were the Kolmogorov–Smirnov test as well as the Shapiro–Wilk test for statistical analysis, using SPSS (Version 27, IBM). The level of significance was set at *p* < 0.05.

### Ethical considerations

This research study obtained ethical clearance from the Faculty of Health Sciences Research Ethics Committee (REC-839-2020) at the University of Johannesburg. Special consideration was given to the right to privacy, confidentiality and anonymity of the study sample. Sportspersons were provided with an information letter of the study and were required to sign a consent form before participating. Sportspersons had the right to withdraw from the research study at any time. However, because of the anonymous nature of the study, once sportspersons submitted the online questionnaire, withdrawal from the study was no longer possible because of the anonymity of the responses.

## Results

**FIGURE 1:**
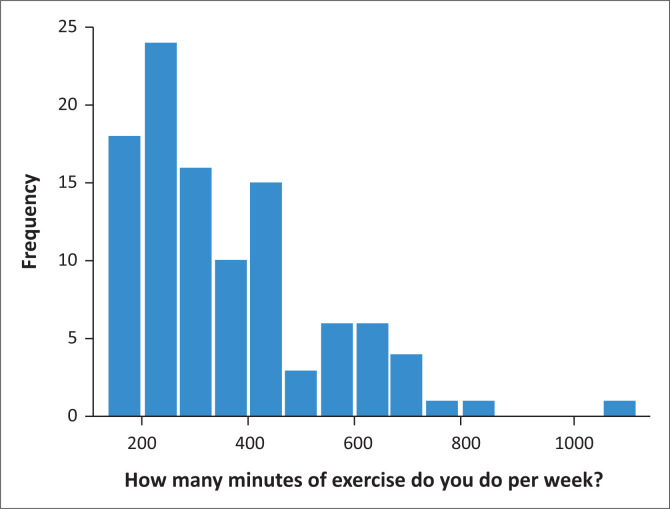
Minutes of exercise performed by each participant per week.

The study sample consisted of both males and females between the ages of 18 and 55. The mean number of exercise minutes achieved per week was 355.10 min and the 95% confidence interval (CI) ranged from 321.45 min per week to 388.74 min per week (*p* = 0.000). Figure 1 presents the number of exercise minutes completed by each participant per week.

**FIGURE 2:**
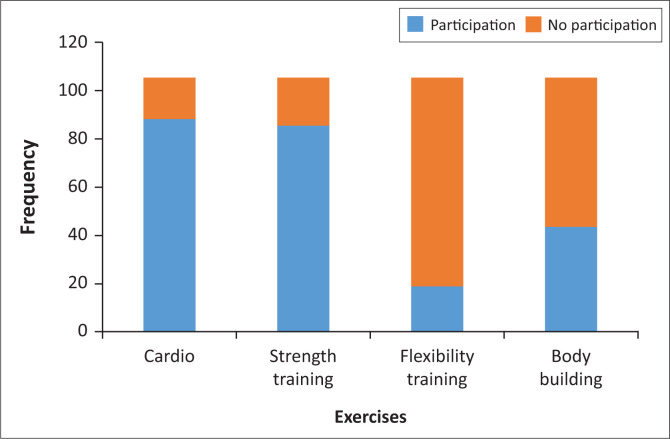
Types of exercise performed.

The type of exercises chosen from the survey included: cardio, strength training, flexibility training and bodybuilding. Cardio training was chosen by the majority of sportspersons (83.8%), whereas flexibility training was chosen by the minority of sportspersons (18.1%) (Figure 2). In addition, the open-ended answers showed that 15.2% of sportspersons played the following sports: soccer, tennis, mountain biking, golf, high intensity interval training (HIIT) exercises, calisthenics, mixed martial arts (MMA), technical boxing, cycling and powerlifting.

**FIGURE 3:**
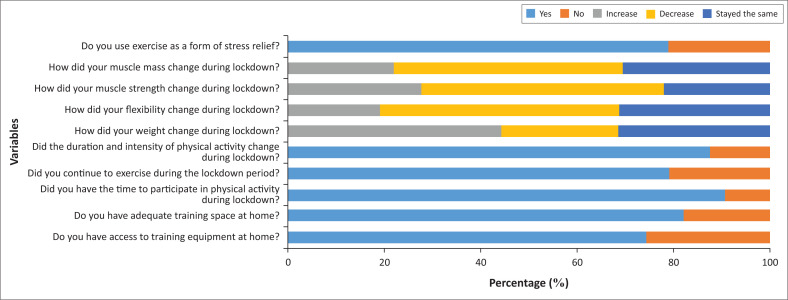
Variables affecting exercise participation.

More than 74% of sportspersons had adequate training space, equipment and the time to perform physical activity and more than 43% of these sportspersons also experienced a decrease in flexibility, muscle mass and muscle strength throughout the duration of lockdown (Figure 3). Exercise was used as a form of stress relief prior to lockdown by 79% of sportspersons and 77.1% of these sportspersons continued to use exercise as a form of stress relief throughout lockdown (Figure 3).

**FIGURE 4:**
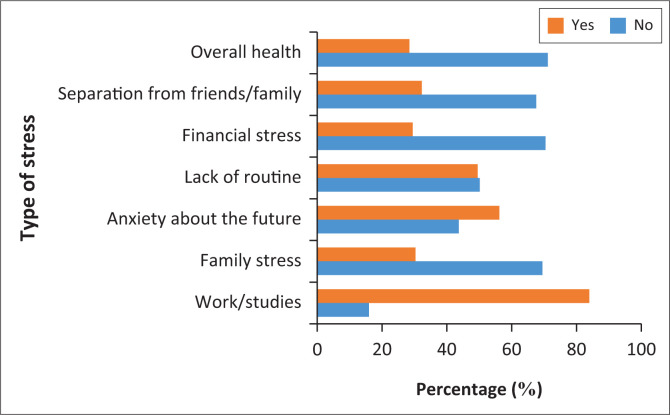
Distribution of stress experienced by each participant.

Other than work or studies, sportspersons experienced family stress, anxiety about the future, lack of routine, financial stress, separation from friends or family and overall health concerns. Sportspersons also experienced stress in the following areas: the closing of training facilities, stress from their relationship and the stress of staying at home (Figure 4).

Of the 35.2% of sportspersons who followed a regular meal plan before the COVID-19 lockdown, 52.5% of sportspersons experienced a change in meal plans during lockdown. Sportspersons expressed that these changes may be because of eating unhealthy foods, which were readily available, such as foods that were high in carbohydrate or experienced a change in taste buds because of being infected with COVID-19. These changes may have attributed to the change in sportspersons’ body weight during lockdown. Additional physical and mental challenges that sportspersons may have experienced during lockdown (that were not reflected here) were feelings of stress, depression, isolation and/or anxiousness. Sportspersons expressed that they may have felt the consequences of inactivity as well as a lack of motivation.

## Discussion

This study examined the relationship between the COVID-19 lockdown on the physical, mental and emotional parameters among regular sportspersons. The main findings from this study were that sportspersons actually participated in cardiovascular training, flexibility training, strength training and bodybuilding exercises during pre-lockdown. During lockdown, more than 74% of sportspersons had adequate training space, equipment and the time to perform physical activity. However, more than 43% of these sportspersons experienced a decrease in flexibility, muscle mass and muscle strength. Exercise was used as a form of stress relief by 77.1% of sportspersons throughout lockdown. In addition, sportspersons who used exercise as a form of stress relief continued to experience an increase in stress throughout lockdown.

### Physical effects

A study conducted by Füzéki, Groneberg and Banzer (2020) concluded that in order to ward off the effects of physical inactivity as well as to strengthen cardiometabolic psychological resources and coping skills, physical activity needs to be implemented on a weekly basis. This study reflected that individuals participated in cardio, strength training, flexibility training and bodybuilding exercises, with majority participating in cardiovascular exercise. This research study has shown that 83 out of 105 sportspersons continued to exercise throughout the lockdown period’ however, they experienced changes in exercise duration and intensity. A majority of sportspersons expressed that they had adequate training space and training equipment at home. One participant stated:

‘Normally when I work out at the gym I train on different machines that intensely works out certain muscles, but at home I worked out with free weights whereby I couldn’t get the full work out for those muscles.’ (Participant 52, 24 years old, participated in 300 minutes of exercise per week)

A positive (yet surprising) finding from this study was that most sportspersons continued to exercise throughout the duration of lockdown even though they had to make significant changes to the intensity and duration of their exercises. Although sportspersons continued to exercise at home, the type of exercises sportspersons performed was not enough to maintain their muscle mass, muscle strength and flexibility. Consequences of a decrease in muscle mass, strength and flexibility lead to deconditioning in sportspersons. Exercises performed in training facilities such as gyms cannot be replicated at home without specific equipment.

The COVID-19 lockdown regulations prevented most sportspersons from going outside to social training areas. Most exercises performed in gyms, swimming pools, tennis courts and training fields cannot be replicated at home. This interfered with the type of exercise that individuals performed at home because of the unavailability of space and gym equipment. A lack of equipment resulted in individuals altering their usual training frequency, intensity, type and duration of their exercises (Roberts, Segovia & Lankford 2019). Athletes who usually train at a higher intensity paired with the newfound stress of the social effects of the coronavirus may have been exposed to developing post-exercise immunosuppression. This placed sportspersons at an increased risk of viral infection, especially one as easily transmitted as COVID-19 (Lim & Pranata 2020).

Fitness influencers who performed gym workouts had to adjust their approach towards home-workout videos (Noonan 2018). These influencers demonstrated how individuals could use alternate household items in place of gym equipment. People were forced to be creative with their gym equipment by replacing workout benches with couches, barbells with heavy water buckets on broomsticks, and resistance bands with shower towels (McClendon 2018). A study in Wroclaw states that home workouts can be just as effective as gym workouts however the exact benefits cannot be replicated (Cortis et al. 2020).

A decline in physical activity over a prolonged period of time resulted in individuals experiencing detraining and deconditioning. This led to a decrease in muscle mass, muscle strength and flexibility leading to an increased risk for joint stiffness, and muscle or joint pain (Piepoli, Dellborg & Börjesson 2019). A decline in physical activity may lead one towards a sedentary lifestyle. A direct effect of a sedentary lifestyle is an increase in the individual’s body mass index (BMI) leading to obesity. A lack of physical exercise is one of the leading causes in preventable death worldwide. A 2018 study carried out by the WHO described 38.2% of all South Africans as insufficiently active.

### Mental and emotional effects

Research has shown that a decrease in social contact and an increase in self-isolation are linked to poor mental health (Ingram, Maciejewski & Hand 2020). The relationship between exercise during lockdown and exercise as a form of stress relief showed a negative impact on sportspersons. In this study, those sportspersons who used exercise as a form of stress relief continued to experience an increase in stress throughout lockdown. Roots of the stress they experienced included work and studies, overall health, separation from family and friends, financial stress, a lack of routine, anxiety about the future and family stress. The uncertainty of lockdown and the effects it has on each individual varies. For example, in this study, some sportspersons used lockdown as motivation to achieve their goals. One participant stated:

‘I took advantage over lockdown in terms of doing additional research about diet plans and work out plans so that once lockdown is over, I already have a game plan to get started again opposed to being lazy because of lockdown. This was actually a form of motivation to get started as lockdown did hinder my body goals slightly.’ (Participant 102, 24 years old, participated in 720 minutes of exercise per week)

According to the WHO, mental health is defined as a state of overall health by which an individual realises his or her own capabilities, the ability to cope with the normal life stressors, the ability to work productively, and the ability to make a contribution to his or her community. The COVID-19 outbreak is a traumatic experience that affected all South Africans. Trauma triggers an emotional health imbalance, which can lead to many physical symptoms, such as stress, anxiety and depression (Sullivan & Robertson 2020). The unpredictability of the new state of living, the loss of lives, the loss of jobs and overall change of lifestyle was distressing to many individuals. Social isolation is the physical distancing from other people, which occurs in the absence of social relationships. Social isolation is imperative to stop the spread of the virus but had a negative impact on the mental health of many individuals. Studies have shown that social isolation causes a feeling of loneliness that results in a decline in cognition and mood, increases the build-up of the stress hormone cortisol, disrupts sleep and functioning of the immune system and therefore results in an increase in body weight (Cudjoe 2020). The individuals who experienced this developed symptoms of depression and anxiety, and may be at risk for cognitive decline and substance abuse.

A study published in the *American Psychologist* investigates the relationship between social relationships and health. The article states that more socially isolated individuals have a lower psychological and physical health and are more likely to die than those who are socially integrated (Scheier & Carver 2018). In the age of digitalisation, going to the gym, eateries, bars, etc. helped us keep social ties with one another. Suddenly, not being able to do that anymore has forced us to adapt to spending time alone with ourselves, our thoughts and our families, which either added further pressure or repaired our relationships. Loneliness does not only affect mental health but physical health as well. Loneliness is shown to be a risk factor of sensory loss, connective tissue and auto-immune disease, cardiovascular disorders and obesity. This then leads to an increase in chronic conditions and a decrease in physical activity. During the lower levels of lockdown, when people were able to go out to buy necessities, it was and still is compulsory to wear a face mask, sanitize your hands and have your temperature taken at every store you visited. The face mask had also affected our social interactions with one another as it decreased our ability to read one another’s emotions and facial expressions.

### Effects on sleep

The literature documents numerous associations of emotional effects on sleep patterns. As previous research shows, the COVID-19 lockdown is associated with changes in sleep patterns as well as the quantity and quality of sleep (Gupta et al. 2020). According to the results of this study, a majority of sportspersons experienced an increase in the amount of sleep during lockdown, whereas the other sportspersons experienced a decrease in the amount of sleep. These sportspersons expressed that these changes were because of having extra time, feeling bored and lazy, having to work and study remotely as a result of no immediate pressure to arrive at work or varsity. Other sportspersons spent this time playing video games and watching television (Netflix) with friends. However, some sportspersons expressed that their stress and anxiety negatively affected their sleeping patterns and social well-being.

### Effects on diet

Aside from sleep patterns, diet can also be affected, mainly driven by emotional fluctuations caused by numerous stressors. A previous study documented that their study sample had experienced an unhealthy change in their diet during lockdown compared with what their diet consisted of during pre-lockdown (Ingram et al. 2020). According to this study, 37 sportspersons followed a regular diet plan during pre-lockdown and 31 out of the 37 sportspersons experienced a change in their regular meal plan during lockdown. Jiang, King and Prinyawiwatkul (2014) associated emotions with eating behaviour as well as an individual’s psychological state with an emotional response to food. Out of 105 sportspersons, 50 sportspersons from this study stated that their emotions had an effect on their diet. One participant who experienced changes in their meal plan during lockdown stated:

‘One of the biggest challenges during lockdown was and still is comfort eating. With your mental state not being healthy you automatically pay less attention to your physical state. Besides that, one of the many perks of being isolated in an Indian household means there’s always something fatty or oily to eat.’ (Participant 100, 22 years old, participated in 680 minutes of exercise per week)

This may have been the cause of a majority of the study sample expressing an increase in weight gain during lockdown (He et al. 2021). Individuals who routinely relied on unhealthy takeaways and who are not well informed about the preparation and portion size of a healthy meal were at a disadvantage during this period. They resorted to eating easily accessible meals that included foods that are high in calories, which contributed to an increase in BMI (D’Isanto, Manna & Altavilla 2017).

## Conclusion

In this research study, it was found that a majority of sportspersons continued to participate in physical activity, even though they experienced significant changes in their muscle mass, flexibility and weight. It is, therefore, imperative to increase measures taken to provide more methods of stress relief during similar periods of lockdown (or exercise restrictions) for those who rely on exercise as their outlet. Stress causes an increase in cortisol, which can also result in an increase in weight gain. The uncertainty of the COVID-19 pandemic, the fear of losing loved ones, financial difficulties, job losses, loneliness and other factors play a major role in one’s mental, emotional and physical health. Additional consequences of the pandemic experienced by most sportspersons in this study included stress, anxiety, depression and insomnia. The extent of the effects of the physical, mental and emotional parameters could have been further explored by the researchers. As a result of physical distancing, anthropometric measurements and objective testing were not possible, which would have optimised our understanding to the extent of changes that sportspersons had experienced during COVID-19.
